# Supplementary material for: Causal Relationship between Aging and Anorexia Nervosa: A White-Matter-Microstructure-Mediated Mendelian Randomization Analysis
Source: Biomedicines. 2024 Aug 16;12(8):1874. doi: 10.3390/biomedicines12081874 (PMC11351342; doi:10.3390/biomedicines12081874)
Supplement: Supplementary file 1 [file biomedicines-12-01874-s001.zip › Supplementary Figure/Figure S4/Singe SNP plot of MR analysis for TL to ubm-b-1778.pdf]

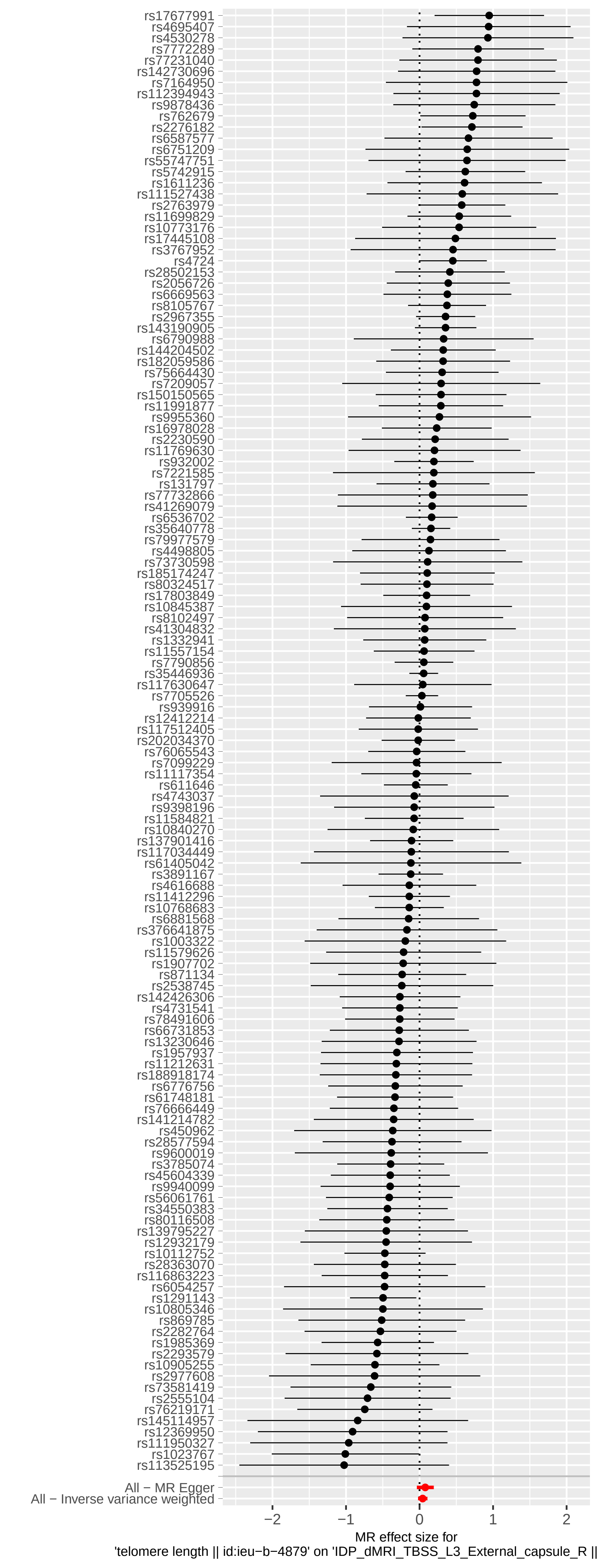
rs17677991
rs4695407
rs4530278
rs7772289
rs77231040
rs142730696
rs7164950
rs112394943
rs9878436
rs762679
rs2276182
rs6587577
rs6751209
rs55747751
rs5742915
rs1611236
rs111527438
rs2763979
rs11699829
rs10773176
rs17445108
rs3767952
rs4724
rs28502153
rs2056726
rs6669563
rs8105767
rs2967355
rs143190905
rs6790988
rs144204502
rs182059586
rs75664430
rs7209057
rs150150565
rs11991877
rs9955360
rs16978028
rs2230590
rs11769630
rs932002
rs7221585
rs131797
rs77732866
rs41269079
rs6536702
rs35640778
rs79977579
rs4498805
rs73730598
rs185174247
rs80324517
rs17803849
rs10845387
rs8102497
rs41304832
rs1332941
rs11557154
rs7790856
rs35446936
rs117630647
rs7705526
rs939916
rs12412214
rs117512405
rs202034370
rs76065543
rs7099229
rs11117354
rs611646
rs4743037
rs9398196
rs11584821
rs10840270
rs137901416
rs117034449
rs61405042
rs3891167
rs4616688
rs11412296
rs10768683
rs6881568
rs376641875
rs1003322
rs11579626
rs1907702
rs871134
rs2538745
rs142426306
rs4731541
rs78491606
rs66731853
rs13230646
rs1957937
rs11212631
rs188918174
rs6776756
rs61748181
rs76666449
rs141214782
rs450962
rs28577594
rs9600019
rs3785074
rs45604339
rs9940099
rs56061761
rs34550383
rs80116508
rs139795227
rs12932179
rs10112752
rs28363070
rs116863223
rs6054257
rs1291143
rs10805346
rs869785
rs2282764
rs1985369
rs2293579
rs10905255
rs2977608
rs73581419
rs2555104
rs76219171
rs145114957
rs12369950
rs111950327
rs1023767
rs113525195
All − MR Egger
All − Inverse variance weighted
−2
−1
0
1
2
MR effect size for
'telomere length || id:ieu−b−4879' on 'IDP_dMRI_TBSS_L3_External_capsule_R ||'
